# A hybrid technique of intracavitary and interstitial brachytherapy for locally advanced cervical cancer: initial outcomes of a single-institute experience

**DOI:** 10.1186/s12885-019-5430-x

**Published:** 2019-03-12

**Authors:** Naoya Murakami, Kazuma Kobayashi, Satoshi Shima, Keisuke Tsuchida, Tairo Kashihara, Nikolaos Tselis, Rei Umezawa, Kana Takahashi, Koji Inaba, Yoshinori Ito, Hiroshi Igaki, Yuko Nakayama, Koji Masui, Ken Yoshida, Tomoyasu Kato, Jun Itami

**Affiliations:** 1 Department of Radiation Oncology, National Cancer Center Hospital, 5-1-1 Tsukiji, Chuo-ku, Tokyo, 104-0045 Japan; 2 Department of Gynecologic Oncology, National Cancer Center Hospital, 5-1-1 Tsukiji, Chuo-ku, Tokyo, 104-0045 Japan; 3 Department of Radiology, Kyoto Prefectural University of Medicine, Kyoto, Japan; 4 Department of Radiology, Osaka Medical College, Takatsuki, Osaka, Japan; 5 Department of Radiotherapy and Oncology, Goethe-University Frankfurt, Frankfurt am Main, Germany

**Keywords:** Uterine cervical cancer, Hybrid of intracavitary and interstitial brachytherapy, Patterns of recurrence, Brachytherapy

## Abstract

**Background:**

Locally advanced uterine cervical cancer (LAUCC) with lateral tumor extension may not always be covered adequately by conventional intracavitary brachytherapy (ICBT). Hybrid intracavitary and interstitial brachytherapy (HBT) seems to be an effective alternative by improving anatomy-oriented dose optimisation. The purpose of this study was to report initial clinical result for LAUCC treated by HBT.

**Methods:**

Between January 2012 and November 2015, 42 patients with LAUCC (T1b2-4a) were treated with primary radiation therapy including HBT. Patients with distant metastasis other than para-aortic lymph node spread were excluded from this study. A retrospective analysis was performed for toxicity evaluation and oncological outcome calculation.

**Results:**

Median follow-up was 23.2 months (range 13.2–71.4). Two-year overall survival, progression free survival, and local control rate were 81.6, 54.4, and 80.2%, respectively. Seven patients experienced local recurrence (16.6%). Of those, five were confined to the uterus and two at the parametria. Late adverse events ≥ grade 3 were seen in 3 patients.

**Conclusions:**

HBT can generate favorable local control in tumors which cannot be adequately covered by ICBT.

## Background

In the primary management of locally advanced uterine cervical cancer (LAUCC) the addition of brachytherapy to external beam radiation therapy (EBRT) with concurrent cisplatin has shown to be associated with higher cause specific- as well as overall survival [1]. Traditionally, intracavitary brachytherapy (ICBT) with tandem plus ovoids/ring was used for irradiation delivering the treatment dose to specific reference points regardless of target size or shape [2]. Although disease control for smaller tumors which can be well-covered by non-anatomy-oriented conventional ICBT dosimetry is encouraging, control rates for large or irregular shaped tumors which are inadequately approached with point A dosimetry are unfavorable [3, 4]. To overcome this challenge, image-guided adaptive brachytherapy (IGABT) [5, 6] with incorporation of a hybrid intracavitary and interstitial brachytherapy technique (HBT) [7–10] has been implemented resulting in markedly improved clinical outcomes [11]. So far, there is no published prospective trial investigating the superiority of HBT over conventional ICBT in the management of LAUCC even though its advantages seem plausible considering that it enables for individualised, three-dimensional (3D) anatomy-oriented dosimetry. However, a phase I/II prospective clinical study is under way testing the feasibility and efficacy of HBT for LAUCC [12].

In our department, HBT has been applied since 2012 and the aim of the current analysis was to report our clinical results after HBT for patients with LAUCC.

## Methods

Uterine cervical cancer patients who underwent primary radiation therapy (RT) for LAUCC incorporating at least one HBT application between January 2012 and November 2015 were included in this single-institutional retrospective study. LAUCC was defined as any tumor with a size or shape which cannot be adequately covered by conventional ICBT isodose distribution. This was determined according to findings derived from weekly performed gynecological examinations and interim magnet resonance imaging (MRI) taken within 1 week before initial brachytherapy. Staging work-up included pelvic examination, cystoscopy, chest/pelvic computed tomography (CT), trans-rectal ultrasound (TRUS), and pelvic MRI. Patients with metastases other than para-aortic lymph nodes were excluded from this analysis. Patient’s demographics are summarised in Table 1.

**Table 1:** Patients’ characteristics (*n* = 42)

Median age (years)		59 (range, 30–85)
Histologic subtypes	Squamous	40
	Adeno	2
T	IB2	3
	IIA2	2
	IIB	5
	IIIA	1
	IIIB	28
	IVA	3
N	0	22
	1	20
Ma		6
Tumor diameter (cm)		6 (range, 3.9–10.1)
Ulceration	Yes	20
	No	22
Hydronephrosis	Yes	10
	No	32
Pyometra	Yes	12
	No	30
Corpus invasion	Yes	26
	No	16

T: primary tumor stage

N: regional lymph nodal stage

Ma: paraaortic lymph node metastasis

### Chemotherapy and external beam radiation therapy

The principles of primary RT of LAUCC in our department are described in detail elsewhere [13, 14]. At this, patients with tumor size larger than 4 cm at initial diagnosis and/or lymph node metastasis are candidates for concurrent chemoradiation therapy (CCRT) with weekly administration of cisplatin (40 mg/m^2^). Patients older than 76 years or without adequate renal function are treated with RT alone.

Radiation therapy was delivered as conventionally fractionated 3D-conformal EBRT by a 4-field box technique using a linear accelerator (Clinac iX or TrueBeam, Varian Medical System, Palo Alto, CA) with 15 MV photons. Treatment planning was based on CT images of 3 mm slice interval (Aquilion™ LB CT scanner, Toshiba Medical System, Japan). The radiation portals covered the whole uterus, parametria, vagina, and the regional lymphatics (pre-sacral nodes, common-, internal-, and external-iliac nodes). The upper and lower margin was set at L4/5 and the lower border of the obturator foramen, respectively. Initially, 30–40 Gy were delivered to the whole pelvis followed by application of an AP-PA central shield (CS) by means of a 4 cm-width block to reduce the dose to the rectum and bladder until the total prescription dose for pelvic side wall of 50 Gy was reached. In cases in which tumor response was very poor, no CS was applied and whole pelvic EBRT was delivered up to 50 Gy. For this purpose, tumor response was assessed every week by physical examination.

### Brachytherapy

Brachytherapy was initiated during the second half of the EBRT course after application of CS with administration of 1–2 single-fraction sessions per week. For this purpose, MRI was performed before the first brachytherapy to confirm the indication for HBT and to aid for high-risk clinical target volume (HR-CTV) contouring [6, 15, 16]. Regarding the treatment devices itself, intracavitary applicators were either tandem/ovoid or tandem/cylinder. Additional interstitial catheters were inserted perineally or vaginally under TRUS guidance [15] with implantation being performed under saddle block anesthesia or local anesthesia and intravenous sedation.

**Fig. 1:**
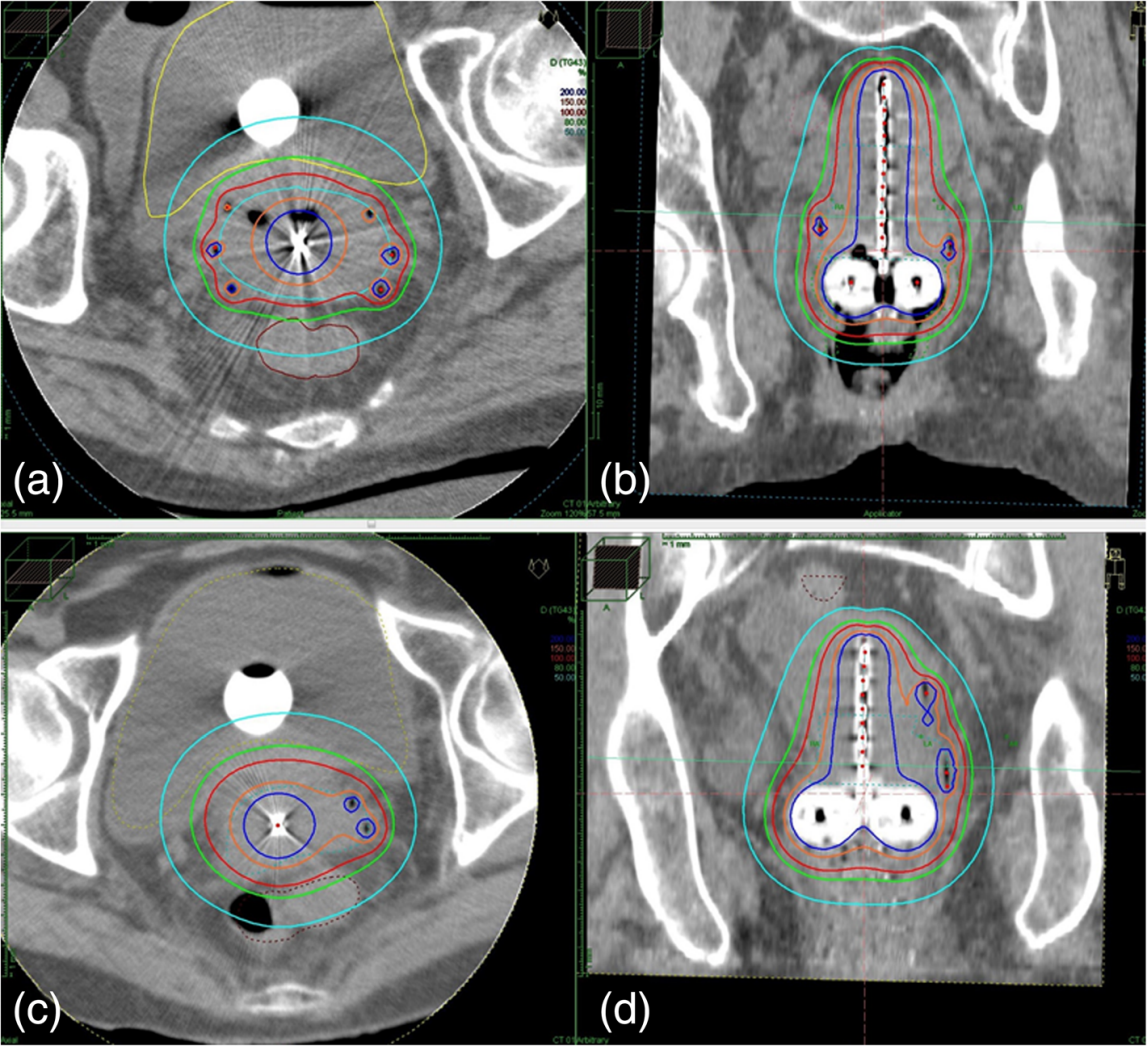
Examples of dose distributions of computed tomography-based hybrid intracavitary and interstitial brachytherapy (HBT). **a** axial and (**b**) coronal images of HBT for a IIIB patient with bilateral parametrial invasion. In each side of the parametrium, three additional interstitial needles were inserted to cover lateral tumor extension. **c** axial and (**d**) coronal image of HBT for a IIIB patient with left parametrial invasion. Two interstitial needles were inserted in the left parametrium

Brachytherapy planning was CT- based (Fig. 1) with a slice thickness of 2 mm generated by a large bore CT simulator (Aquilion™, Toshiba, Tokyo, Japan). Planning imaging was performed in lithotomy position with the applicators in situ and treatment planning was executed using the planning software Oncentra® (Elekta, Veennendaal, The Netherlands). High-risk clinical target volume (HR-CTV) was not defined MRI-based [5, 6] because it was not possible to generate MRI for every single-fraction session but contoured based on CT imaging [12, 17, 18]. However, pre-interventional MRI was used as reference. All treatments were carried out by a ^192^Iridium remote after loading system (RALS, MicroSelectron HDR™, Elekta, Veennendaal, The Netherlands).

The dosimetric goal was to deliver more than 6 Gy to the HR-CTV D_90_ (dose covering at least 90% of the HR-CTV) while keeping doses to organs at risk (OARs) as low as possible. In some cases, if favorable tumor shrinkage was obtained after initial HBT, brachytherapy was completed with ICBT without additional interstitial catheter implantation.

### Dose calculation and Dosimetric indices

In summing the dose of EBRT and HBT, the equivalent dose in 2 Gy fractions (EQD_2_) [6] according to the LQ model [19, 20] was calculated according to the following formula:

$$ \mathrm{EQD}2=\frac{\mathrm{Nd}\left(1+\frac{d}{\upalpha /\upbeta}\right)}{1+\frac{2}{\upalpha /\upbeta}} $$

The parameter *N* indicates the number of fractions and *d* the dose per fraction. For calculating tumor and OARs doses, α/β was assumed as 10 Gy and 3 Gy, respectively. Dose contribution from CS was not taken into consideration [21].

The minimum dose covering 90% of the HR-CTV (HR-CTV D_90_) in EQD_2_ was used as the representative dose of HBT. In order to analyse further the relationship between dose distribution and local control (LC), an additional CTV-Corpus was defined as follows: HR-CTV was divided into two parts by the most cranial level of uterine cervix and the volume cranial to the level was defined as CTV-Corpus. Because no contrast enhancement agent was used in simulation CT imaging, the point at which the uterine volume expands was used as surrogate structure of the upper limit of uterine cervix. CTV-Corpus V_100_ (percentage of CTV-Corpus covered by 100% of the prescription dose) at the first brachytherapy was measured and its relation to LC, PSF, and OS was analysed.

### Follow-up

All patients were evaluated weekly for acute adverse events during RT by physical examination and blood tests. Computed tomography and/or MRI and cytology were performed 3 months after completion of RT to evaluate initial tumor response and imaging was repeated every 3–6 months for the first 5 years and annually thereafter.

### Statistical analysis

Overall survival (OS) was estimated from the start of RT to the date of death from any cause or censored at the last follow-up visit. Progression-free survival (PFS) was estimated from the start of RT to the date of any disease relapse or censored at the last follow-up visit. For LC calculation, central and parametrial relapse was considered as an event. Local control was estimated from the start of RT to the date of local relapse or censored at the last follow-up. Survival curves were estimated by using the Kaplan-Meier method and the differences were assessed by the log-rank test. A *p* value ≤0.05 was considered statistically significant. Factors with *p* value ≤0.05 were further analysed in the multivariate analysis by the Cox regression method. Cox proportional-hazards models were used to estimate hazard ratios. All statistical analyses were performed using IBM SPSS Statistics (version 18.0; SPSS, Inc., Chicago, IL).

This retrospective study was also approved by the Institutional Review Board of our hospital, the Ethics Committee of National Cancer Center Hospital, (approval number is 2015–359) according to the ethical standards laid down in the Declaration of Helsinki. Written informed consent was taken from all the participants included in this study before treatment.

## Results

From January 2012 to November 2015, 42 consecutive uterine cervical cancer patients with histologically proven squamous cell carcinoma or adenocarcinoma were treated with definitive RT including HBT. Median follow-up for patients who were still alive at last follow-up visit was 23.2 months (range 13.2–71.4).

Treatment details are summarized in Table 2. Median volume of HR-CTV at initial HBT measured by CT was 37.1 ml (range 12.1–89.2 ml). The median number of brachytherapy fractions was 4 (range 2–5). Median dose of HR-CTV D_90_ in EQD_2_ was 70.3 Gy (range 56.2–97.3 Gy).

**Table 2:** Treatment details, *n* = 42

		*N* = 42
Median dose prescribed to the whole pelvis (Gy)		30.6 (range, 26–50.4)
Median brachytherapy fractions (n)		4 (range, 2–5)
Median volume of HR-CTV at initial HBT (ml)		37.1 (range, 12.1–89.2)
Median HR-CTV D_90_ in EQD_2_ (Gy)		70.3 (range, 56.2–97.3)
Median HR-CTV V_100 (%)_		95.9 (range, 79.3–100)
Median HR-CTV V_150 (%)_		57.7 (range, 39.1–89.4)
Median HR-CTV V_200 (%)_		29.9 (range, 19.6–58.5)
Median dose of bladder D_2cc_ in EQD_2_ (Gy)		72.4 (range, 60.7–87.2)
Median dose of rectum D_2cc_ in EQD_2_ (Gy)		64.5 (range, 47.6–75.2)
Concurrent chemotherapy	Yes	28
	No	14

**Fig. 2:**
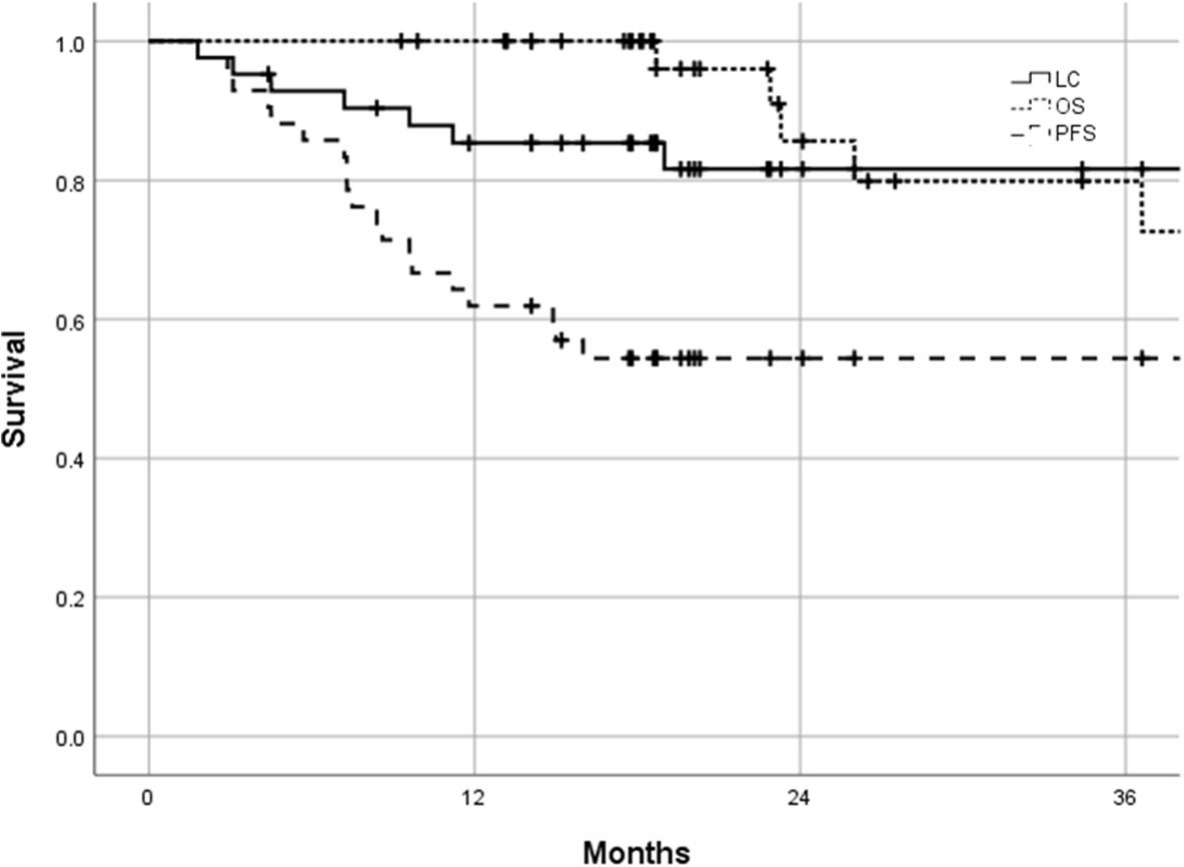
Kaplan-Meier survival curves for local control (LC), progression-free survival (PFS), and overall survival (OS) of LAUCC patients treated by computed tomography-based hybrid intracavitary and interstitial brachytherapy

The 2-year OS, PFS, and LC were 81.6, 54.4, and 80.2%, respectively (Fig. 2). Nineteen patients experienced disease relapse with eleven patients having loco-regional failure and 15 patients distant metastasis. Of the seven patients with local recurrence (16.6%), in five the failure site was the uterus and in two the parametria.

**Fig. 3:**
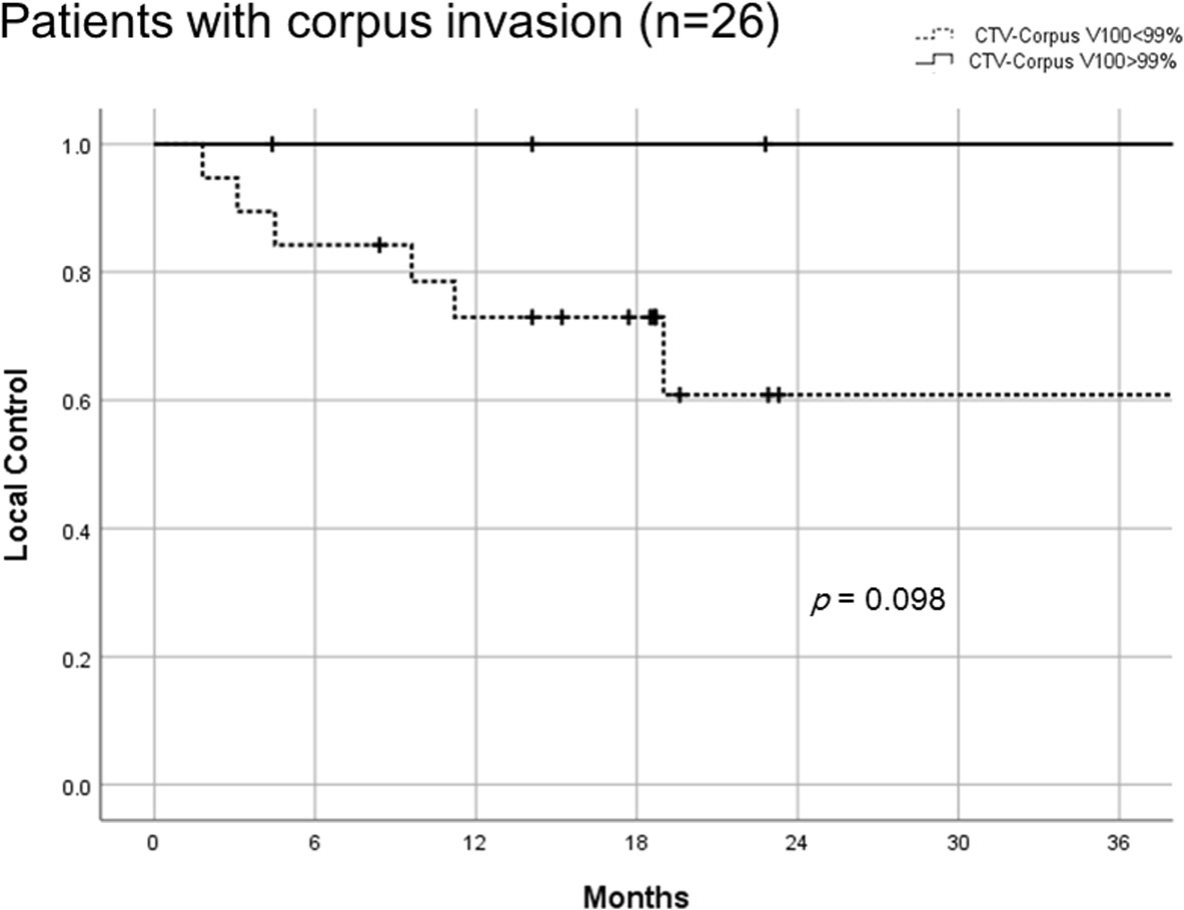
Kaplan-Meier survival curves for local control (LC) for patients with corpus invasion (*n* = 26) stratified by CTV-Corpus V_100_

Factors potentially correlating with LC, PFS, or OS were analysed and summarised in Table 3. Factors showing statistical significance regarding LC were initial tumor size, radiologic feature of both ulceration and corpus invasion, HR-CTV volume at first brachytherapy, and mean HR-CTV V_200_ (percentage of the HR-CTV receiving at least 200% of the prescription dose). Tumors whose HR-CTV volume at first brachytherapy was < 40 ml had better LC than HR-CTV volume > 40 ml (2-y LC 95.5% vs 71.2%, *p* = 0.032). Tumors with mean HR-CTV V_200_ > 34% had better LC than mean HR-CTV V_200_ < 34% (2-y LC 100% vs 76.4%, *p* = 0.042). A trend for improved LC was seen in patients with CTV-Corpus V_100_ > 99% vs. CTV-Corpus V_100_ < 99% (2-y LC 100% vs 73.4%, *p* = 0.062). Similarly, in sub-group analysis of patients with corpus invasion (*n* = 26) a trend for improved LC was seen in cases with CTV-Corpus V_100_ > 99% vs. CTV-Corpus V_100_ < 99% (2-y LC 100% vs 60.8%, *p* = 0.098, Fig. 3). In multi-variate analysis, patients showing radiologic features of both ulceration and corpus invasion had worse LC, PFS, and OS (Table 3). In addition, initial tumor size > 6 cm was also found out to be an adverse factor for PFS in multi-variate analysis.

**Table 3:** Potential predictors influencing local control (LC), progression-free survival (PFS), and overall survival (OS)

	2-y LC (%)		LC		2-y PFS (%)		PFS		2-y OS (%)		OS	
	yes (n)	no (n)	*p* value in uni.	*p* value in multi.	yes	no	*p* value in uni.	*p* value in multi.	yes	no	*p* value in uni.	*p* value in multi.
Scc vs Non-Scc	86.0 (40)	50 (2)	0.101		54.6 (40)	50 (2)	0.967		79.9 (40)	100 (2)	0.669	
LN+	89.5 (20)	78.8 (22)	0.878		40.0 (20)	67.5 (22)	0.052		73.5 (20)	84.5 (22)	0.448	
Initial tumor size > 6 cm	73.2 (21)	95.2 (21)	0.042^*^		33.3 (21)	75.9 (21)	0.005^*^	*p* = 0.043, HR 0.333, 95%CI 0.115–0.965	75.3 (21)	85.0 (21)	0.037^*^	
Ulceration plus corpus invasion	56.4 (15)	96.3 (27)	0.006^*^	*p* = 0.017, HR 0.123, 95%CI 0.22–0.689	26.7 (15)	70.0 (27)	0.002^*^	*p* = 0.027, HR 0.340, 95%CI 0.131–0.882	46.7 (15)	96.2 (27)	0.001^*^	*p* = 0.003, HR 0.118, 95%CI 0.029–0.482
Hydronephrosis	77.1 (10)	87.1 (32)	0.960		50.0 (10)	56.1 (32)	0.981		88.9 (10)	76.9 (32)	0.316	
Pyometra	91.7 (12)	81.4 (30)	0.433		50.0 (12)	56.3 (30)	0.554		68.8 (12)	84.1 (30)	0.972	
Combination of weekly CDDP	80.3 (28)	92.3 (14)	0.706		56.9 (28)	49.0 (14)	0.917		81.8 (28)	76.6 (14)	0.968	
HR-CTV at initial BT > 40 ml	71.2 (19)	95.5 (23)	0.032^*^		36.8 (19)	69.1 (23)	0.026^*^		74.2 (19)	86.7 (23)	0.297	
HR-CTV D_90_ > 70 Gy	85.2 (24)	83.0 (18)	0.888		45.5 (24)	66.7 (18)	0.156		83.5 (24)	76.2 (18)	0.820	
CTV-Corpus V_100_ > 99%	100 (13)	73.4 (29)	0.062		59.8 (13)	51.7 (29)	0.457		83.9 (13)	76.8 (29)	0.412	
Mean HR-CTV V_200_ > 34%	100 (14)	76.4 (28)	0.042^*^		55.6 (14)	53.6 (28)	0.699		85.1 (14)	77.2 (28)	0.396	

*PFS* progression-free survival, *OS* overall survival, uni.: univariate analysis, multi.: multivariate analysis, *LN+* regional lymph node positive, *HR-CTV* high risk clinical target volume, *BT* brachytherapy, *HR-CTV D*_*90*_ minimum dose covering 90% of the HR-CTV, *HR-CTV V*_*200*_ the percentage of the HR-CTV receiving higher than 200% of the prescribed dose

Late adverse events ≥ grade 3 were seen in 3 patients; one patient suffered from grade 3 rectal bleeding and ileus requiring hyperbolic oxygen therapy (HBO) and ileostomy, one experienced grade 3 rectal bleeding and vaginal ulcer requiring HBO, and one developed rectovaginal fistula. Rectum D_2cc_ for those three patients were 66.7 Gy, 68.1 Gy, and 67.5 Gy, respectively. Patient with vaginal ulcer had extensive vaginal invasion, therefore, she was treated with tandem, cylinder, and additional interstitial needles in the brachytherapy. The vaginal ulcer was developed circumferentially in her upper third of vagina. Vaginal D_2cc_ for this patient was 157.3Gy which was higher than 145 Gy which was found to be a threshold for developing vaginal ulcer in our previous study [22].

## Discussion

In this study the initial oncological results of LAUCC patients receiving primary RT including HBT were promising and consistent with other published experiences. Even though pretreatment median tumor diameter was 6 cm, a 2-y LC of 80.2% could be achieved despite the fact that tumor size represents a known adverse prognostic factor for primary RT in cervical cancer. At this, Toita et al. reported on a phase II multi-institutional prospective clinical trial using ICBT as mode of brachytherapy in patients with LAUCC generating a 2-year pelvic disease progression-free rate (PDPF) of 72% for tumors of 5–7 cm and of 54% for tumors larger than 7 cm [4]. Likewise, Pötter et al. reported a 2-y PDPF rate of 64% for patients treated by EBRT plus ICBT and whose initial tumor size was larger than 5 cm [3]. Therefore it may be concluded that the 2-y LC of 80% in our study was better than clinical outcomes associated with conventional ICBT for LAUCC. However, our yielded LC was not satisfactory compared to the other published HBT series reporting LC in the range of 90–95% [11, 23]. On the other hand, the 3-y LC for LAUCC patients who were treated in our institution with multi-catheter HDR interstitial brachytherapy (ISBT) was 87.8% in spite of having larger tumor than in the present study [24]. In this sense, it is conceivable that multi-catheter ISBT allows for more conformal tumor coverage than with HBT, thereby improving target volume dose escalation and subsequently LC. The drawback of multi-catheter ISBT, however, is that within a multi-fraction scheme it is logistically demanding and arduous for the patient requiring confinement to bed during multi-day treatments [25].

The actual indication of ICBT, HBT, and multi-catheter ISBT is a challenging topic which needs clinical experience in order to select the most appropriate brachytherapy modality depending on tumor size and shape [26]. Whatsoever, in our study lateral tumor extent was well controlled with HBT. On the other hand, uterine corpus recurrence was frequently observed in patients with local disease relapse. Although it did not reach statistical significance because of the small patient number analysed, CTV-Corpus V_100_ > 99% was associated with a trend towards better LC (Table 3). In that regard, corpus invasion results in an irregular shape of the uterine corpus potentially prohibiting the adequate position of the tandem. Therefore, extra intra-corpus interstitial catheters may be necessary to adequately cover extensive tumor extent.

In the current study, the median HR-CTV D_90_ was 70.3 Gy EQD_2_ which is lower than the 87 Gy recommended by Dimopoulos et al. [23] and the American Brachytherapy Society (ABS) guidelines [27, 28]. However, HR-CTV D_90_ calculated in this study was derived from the simple addition of the whole pelvis RT dose and HBT while dose contribution from CS was completely ignored according to the Japan Society of Gynecologic Oncology guidelines [21]. At this, Tamaki et al. recently demonstrated that the HR-CTV D_90_ generated by the combination of EBRT and ICBT in consideration of the dose contribution from CS is much higher than without counting for CS [29, 30]. Accordingly, the “corrected” median HR-CTV D_90_ in our series must be estimated greater than 70.3 Gy when ignoring respective national practice guidelines [21].

Concerning the impact of potential prognostic factors, parameters related to LC were initial tumor size > 6 cm, HR-CTV volume > 40 ml and mean HR-CTV V_200_ > 34%. In addition, multivariate analysis showed that having a morphological feature of both ulceration and corpus invasion had also a significant adverse impact (Table 3). A resembling result was depicted in the EMBRACE study indicating that infiltrative tumor growth responded unfavorably to CCRT when compared to expansive tumors [31]. Furthermore, we could show that tumors receiving mean HR-CTV V_200_ > 34% had improved LC. So far, no special attention has been paid in IGABT to the topic of high dose volumes receiving more than 100% of the prescribed reference dose with regard to outcomes correlation. Hereunto, the inherently non-homogeneous dose distribution in brachytherapy performs simultaneous intratumoral dose-boosting with no upper dose limits and a very sharp dose fall-off gradient outside the target volume [32]. The latter is of particular importance as it facilitates the application of very high doses to central tumor areas which might experience increased radioresistance due to hypoxic tumor microenvironment. This dose escalation might be an effective approach to counteract the negative impact of tumor hypoxia as prognostic factor for survival in patients with uterine cervical cancer [33].

One patient experienced development of vaginal ulcer which is relatively rare late radiation-related adverse effect. This patient had extensive vaginal invasion which required tandem and cylinder to cover the target volume in the brachytherapy. When patients have their disease in the vaginal wall, it is difficult to distinguish between pure vaginal ulcer or ulcer-like necrotic tumor shrinkage. On the other hand, vaginal wall D_2cc_ of this patient was 157.3 Gy which was higher than a previously reported threshold of 145 Gy for late vaginal ulcer [22].

There are several limitations in this study. Firstly, HR-CTV was contoured based on CT imaging and might therefore be larger than when defined by MRI [17]. As a result, the comparison with data generated by MRI-based treatment planning must be done cautiously. In addition, our dose schedule was different from the standard dose schedule of the GEC-ESTRO [6] or ABS guidelines [27, 28] not allowing an unconditional extrapolation of the clinical outcome data when using different dose schedules. Finally, this is a retrospective study from a single institution with a limited number of patients. Further investigations are warranted to verify the findings demonstrated in this analysis.

## Conclusion

For locally advanced uterine cervical cancer patients, HBT can provide favorable LC associated with an acceptable late toxicity profile.

## Abbreviations

3D: Three-dimensional
ABS: The American Brachytherapy Society
CCRT: Concurrent chemoradiation therapy
CS: Central shield
CT: Computed tomography
CTV-Corpus V_100_: Percentage of CTV-Corpus covered by 100% of the prescription dose
EBRT: External beam radiation therapy
EQD_2_: The equivalent dose in 2 Gy fractions
HBO: Hyperbolic oxygen therapy
HBT: Hybrid intracavitary and interstitial brachytherapy
HR-CTV D_90_: The minimum dose covering 90% of the HR-CTV
HR-CTV: High-risk clinical target volume
ICBT: Intracavitary brachytherapy
IGABT: Image-guided adaptive brachytherapy
ISBT: Interstitial brachytherapy
LAUCC: Locally advanced uterine cervical cancer
LC: Local control
MRI: Magnet resonance imaging
OS: Overall survival
PDPF: Pelvic disease progression-free rate
PSF: Progression-free survival
RT: Radiation therapy
TRUS: Trans-rectal ultrasound
